# Aberrant resting-state connectivity of auditory, ventral attention/salience and default-mode networks in adults with attention deficit hyperactivity disorder

**DOI:** 10.3389/fnins.2022.972730

**Published:** 2022-09-06

**Authors:** Rina Blomberg, Carine Signoret, Henrik Danielsson, Irene Perini, Jerker Rönnberg, Andrea Johansson Capusan

**Affiliations:** ^1^Department of Behavioral Sciences and Learning, Linköping University, Linköping, Sweden; ^2^Linnaeus Center HEAD, Linköping University, Linköping, Sweden; ^3^Swedish Institute for Disability Research, Linköping University, Linköping, Sweden; ^4^Center for Social and Affective Neuroscience, Department of Biomedical and Clinical Sciences, Linköping University, Linköping, Sweden; ^5^Center for Medical Image Science and Visualization, Linköping University, Linköping, Sweden; ^6^Department of Psychiatry and Biomedical and Clinical Sciences, Linköping University, Linköping, Sweden

**Keywords:** attention deficit hyperactivity disorder, adults, resting state, functional connectivity, default mode network, salience network, auditory network

## Abstract

**Background:**

Numerous resting-state studies on attention deficit hyperactivity disorder (ADHD) have reported aberrant functional connectivity (FC) between the default-mode network (DMN) and the ventral attention/salience network (VA/SN). This finding has commonly been interpreted as an index of poorer DMN regulation associated with symptoms of mind wandering in ADHD literature. However, a competing perspective suggests that dysfunctional organization of the DMN and VA/SN may additionally index increased sensitivity to the external environment. The goal of the current study was to test this latter perspective in relation to auditory distraction by investigating whether ADHD-adults exhibit aberrant FC between DMN, VA/SN, and auditory networks.

**Methods:**

Twelve minutes of resting-state fMRI data was collected from two adult groups: ADHD (*n* = 17) and controls (*n* = 17); from which the FC between predefined regions comprising the DMN, VA/SN, and auditory networks were analyzed.

**Results:**

A weaker anticorrelation between the VA/SN and DMN was observed in ADHD. DMN and VA/SN hubs also exhibited aberrant FC with the auditory network in ADHD. Additionally, participants who displayed a stronger anticorrelation between the VA/SN and auditory network at rest, also performed better on a cognitively demanding behavioral task that involved ignoring a distracting auditory stimulus.

**Conclusion:**

Results are consistent with the hypothesis that auditory distraction in ADHD is linked to aberrant interactions between DMN, VA/SN, and auditory systems. Our findings support models that implicate dysfunctional organization of the DMN and VA/SN in the disorder and encourage more research into sensory interactions with these major networks.

## Introduction

**FIGURE 1:**
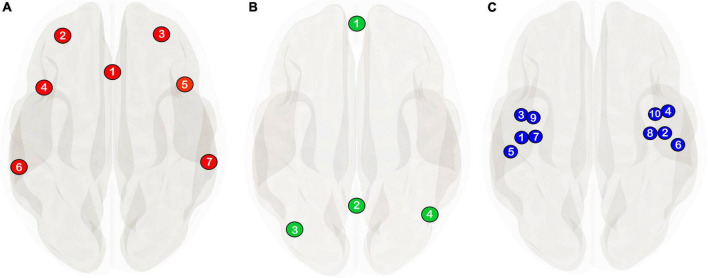
The network delineations used in the current study are from the CONN-toolbox’s v.20b (Whitfield-Gabrieli and Nieto-Castanon, 2012; Nieto-Castanon, 2020) network atlas derived from an independent component analysis of 497 individuals from the human connectome project. **(A)** Ventral attention/salience network (see Uddin et al., 2019 for a discussion on network nomenclature) consisting of the anterior cingulate (1) and bilateral rostral prefrontal (2, 3), anterior insula (4, 5) and supramarginal cortices (6, 7). **(B)** Default-mode network definition comprising of the medial prefrontal cortex (1), the posterior cingulate cortex, (2) and bilateral angular gyri (3, 4). **(C)** Auditory seed regions used in the current study are the same anatomically defined regions of interest from Blomberg et al. (2021) comprising of the bilateral Heschl’s gyrus (1, 2), planum polare (3, 4), planum temporale (5, 6), granular posterior insulae (7, 8), and dysgranular posterior insulae (9, 10).

Current etiological models of attention deficit hyperactivity disorder (ADHD) emphasize dysfunctional interactions between intrinsic brain networks, rather than regional brain abnormalities, for explanations of behavioral and clinical symptoms in the disorder (for reviews see: Konrad and Eickhoff, 2010; Castellanos and Proal, 2012; Posner et al., 2014; Castellanos and Aoki, 2016; Sutcubasi et al., 2020). Two particular intrinsic brain networks: the default-mode network (DMN; Figure 1A), and the ventral attention/salience network (VA/SN; Figure 1B); are hypothesized to play a pivotal role in clinical aspects of inattention in ADHD. The DMN is generally more active when attention is directed internally—i.e., to introspective, self-referential thought in the absence of stimulus-driven tasks. The VA/SN is more active when attention is directed externally, and is heavily implicated in the vigilant anticipation, detection, and response-mediation of behaviorally salient stimuli. In healthy individuals, the DMN and VA/SN show robust anticorrelated (i.e., phasically negatively correlated) functional connectivity (FC) at rest (Anticevic et al., 2012). And this anticorrelated relationship is considered an inherent representation of the opposing resource demands and attentional states between these networks during goal directed cognitive tasks (Fox et al., 2005, 2009). A variety of resting-state fMRI studies on ADHD have reported a weaker anticorrelated relationship between core DMN and VA/SN regions compared to controls (e.g., Castellanos et al., 2008; Sun et al., 2012; Sripada et al., 2014; Lin et al., 2018; Mills et al., 2018; c.f. also Sato et al., 2012). And these findings have had a notable influence over our conceptualization of inattention in the disorder.

One prevailing perspective for instance suggests that a weaker anticorrelation between core regions of the DMN and VA/SN at rest is an index of poorer DMN regulatory capacity associated with symptoms of mind wandering in the disorder (see Posner et al., 2014 for a review). The logic here being that the DMN is more active during introspective, task-unrelated thought and suppressed during stimulus driven tasks. Hence observations of aberrant resting-state anticorrelation is thought to reflect an inherent susceptibility for attentional lapses resulting from obtruding interoceptive thought (Castellanos et al., 2008; Bozhilova et al., 2018). Alternative perspectives have suggested that the VA/SN plays a pivotal role in the manifestation of attentional deficits in the disorder. Aboitiz et al. (2014) for instance, hypothesized that a loss of anticorrelation impacts the sensitivity of the VA/SN which can lead to a bias toward irrelevant/salient stimuli and therein an increased susceptibility to environmental distraction. Similarly, Menon (2011) proposed that aberrant intrinsic VA/SN organization can result in the inappropriate assignment of saliency to either exogenous stimuli or internal mental events—underpinning, *inter alia*, clinical aspects of inattention in many psychiatric and neurological disorders, including ADHD. Hence, aside from mind wandering, a disrupted antagonistic balance between the DMN and VA/SN in the disorder may also be indicative of an increased sensitivity to the external environment, and it is this notion which founds the basis of inquiry for the current study.

Heightened sensory sensitivity is a frequently reported symptom in adults with ADHD particularly in the auditory modality (Schulze et al., 2020). However, very few resting-state analyses have studied auditory network FC in ADHD-adults. Hence little is known as to whether deficits in auditory attention are associable to the aberrant, intrinsic FC of the DMN and VA/SN implicated in the disorder (Castellanos and Aoki, 2016). Following through on previous work, the current study provides us with a unique opportunity to explore resting-state FC in relation to auditory distraction in this patient group. Here we analyze resting-state data from the same sample of adult participants (ADHD and healthy controls) from Blomberg et al.’s (2021) task-based fMRI study that utilized a cross modal attention paradigm to analyze the effects of working memory load on auditory distraction in adults with ADHD. The paradigm involved two main tasks: an auditory task where the goal was to actively detect salient oddball tones amidst a stream of standard tones, and a visual *n*-back task consisting of 0-, 1-, and 2-back working memory conditions. In all working memory conditions, participants were instructed to detect the visual *n*-back target and ignore the streaming tonal signal from the auditory task, which continued to play. When participants’ attention was focused on the auditory task, auditory cortical activity was enhanced relative to a resting baseline; and when attention was instead directed toward the visual task, auditory processing was attenuated. The degree of attenuation in auditory regions was relative to the cognitive demands of the visual task—the greater the working memory load and attentional engagement in the visual modality, the greater the neural attenuation to task-irrelevant sound in auditory regions. Importantly, for ADHD participants, the relationship between attentional engagement and auditory attenuation proved less efficient than controls. In particular, under the most cognitively demanding visual condition, the 2-back task (2-bT), ADHD participants showed significantly poorer attenuation in auditory regions. Further, this heightened auditory activity was correlated with individual differences in symptomatic inattentiveness and 2-bT performance—for which the ADHD group were significantly inferior to controls. In the current study, we pose the question as to whether these aforementioned outcomes are related to both individual, and group level variances in intrinsic FC between the DMN, VA/SN, and auditory regions.

Our first objective (O1) in this regard was to determine if a weakener DMN–VA/SN anticorrelation was evident in the ADHD group. Our second objective (O2) was to test for group differences in FC between auditory seeds (Figure 1C) and regions pertaining to the VA/SN and DMN. Given the functional antagonism of the VA/SN and DMN with respect to externally and internally directed attention, here we hypothesized that ADHD participants would show increased FC between auditory seeds and VA/SN regions and reduced anticorrelated FC between auditory seeds and DMN regions. Our third objective (O3) was to explore individual differences in brain-behavioral relationships between DMN, VA/SN, and auditory FC. Here the goal was to determine if ADHD-symptom severity and performance on the 2b-T from Blomberg et al. (2021) was associated with increases/decreases in DMN, VA/SN and auditory FC.

## Materials and methods

### Participants

The resting-state data for this study was obtained from the sample of participants from Blomberg et al. (2021) which included 17 clinically stable adults with ADHD (age: *M* = 28, *SD* = 6.8) and 17 healthy controls (age: *M* = 25, *SD* = 5.1). Of the 17 ADHD participants, 15 were prescribed stimulant medication and abstained from their medication for at least 48 h prior to testing. The 18-item adult ADHD self-report scale (ASRS) v.1.1 (Kessler et al., 2005; Rodriguez et al., 2007) was used to assess ADHD-symptom severity associated with inattention, hyperactivity/impulsivity, and combined subtypes in both groups. See Supplementary Methods for further demographic and clinical details of the sample.

### Image acquisition and preprocessing

Immediately prior to the task-based scan published in Blomberg et al. (2021), a ∼12 min, eyes-closed resting-state scan and an anatomical scan was acquired (Siemens Prisma 3T scanner). The functional resting state scan consisted of 940 echo planar imaging (EPI) whole-brain volumes (TR = 761 ms; TE = 24 ms; FA = 53°; FOV = 204 mm × 204 mm; acquisition matrix = 68 × 68; no. of slices = 45; slice thickness = 3 mm; voxel size = 3 mm × 3 mm × 3 mm). The anatomical scan consisted of 3D, T1-weighted MPRAGE (magnetization prepared rapid gradient echo) images (TR = 2300 ms; TE = 2.36 ms; FA = 8°; FOV = 250 mm × 250 mm × 225 mm; acquisition matrix = 288 × 288 × 208; slice orientation = sagittal; slice thickness = 0.9 mm; no. of slices = 208; voxel size = 0.87 mm × 0.68 mm × 0.9 mm) and double-echo spoiled gradient echo sequence field maps (TR = 520 ms; TE = 4.92/7.38 ms; FA = 60°; total EPI readout time = 16.415 ms; blip direction = 1).

Participants were instructed to lie as still as possible, let their mind’s wander, and not to fall asleep. In order to make the acoustic environment as quiet as possible, the external auditory meatus of each ear was first protected with a self-hardening, moldable wax; next, participants were fitted with active noise canceling headphones (OptoAcoustics Ltd., Tel Aviv, Israel) which further attenuated the background EPI gradient noise to ∼58 dB SPL. The headphones were kept in place via inflatable positioning pads (Pearltec MRI/CT Multipad Plus, MagMedix, MA, United States) that also worked to minimize head movements within the 64-channel head coil.

Preprocessing was performed in MATLAB R2020B software using the CONN toolbox v.20.b (Whitfield-Gabrieli and Nieto-Castanon, 2012, 2017)^1^ and included an additional denoising procedure in order to remove confounds of physiological noise (e.g., cerebral white matter, ventricles, large vessels, and cerebrospinal areas), head movement, outlier scans, as well as constant, and first-order linear session effects. We applied the software’s default preprocessing pipeline for volume-based analyses but with indirect normalization to standard stereotactic (MNI) space as we had obtained gradient field maps during image acquisition (Nieto-Castanon, 2020). This particular procedure included: functional realignment and unwarp with the use of fieldmaps for susceptibility distortion correction; slice-timing correction; outlier identification in which framewise displacements greater than 0.9 mm or global BOLD signal changes above five SD were flagged as potential outliers; indirect segmentation and normalization; and spatial smoothing with CONN toolbox’s default Gaussian kernel recommendation of 8 mm FWHM (full width half maximum).

The denoising pipeline consisted of the following two steps:

Nuisance covariates derived from CONN-toolbox’s implementation of anatomical component-based correction (aCompCor) were entered into an ordinary least squares regression in order to remove confounding effects on the estimated BOLD signal in each voxel per subject and run. The covariates included five noise components from cerebral white matter; five noise components from cerebrospinal areas; 12 subject motion components (three translation, three rotation, and their first-order temporal derivatives), outlier scans identified in the preprocessing procedure and components representing the effect of each task-condition convolved with the canonical hemodynamic response function in order to reduce the influence of slow trends, initial magnetization transients as well as constant task-related effects.
Temporal band pass filtering (high pass: 0.008 Hz, low pass: 0.09 Hz) on the BOLD signal was applied in order to minimize the influence of physiological head motion and other noise sources.

Subsequent quality control analysis of preprocessing outcomes indicated that the mean framewise displacement (disregarding outlier scans) associated with micro-head movements (Controls: *M* = 0.08, *SD* = 0.02; ADHD: *M* = 0.10, *SD* = 0.03) was not significantly different between groups, *F*_(1, 32)_ = 3.8, *p* = 0.06; nor was the mean number of valid (i.e., non-outlier) scans (Controls: *M* = 916, *SD* = 14 ADHD: *M* = 912, *SD* = 17) significantly different between groups *F*_(1, 32)_ = 0.5, *p* = 0.49.

### Statistical analysis

All statistical analyses were performed in the CONN-toolbox. The denoised, voxel-wise BOLD time series data was first averaged within each auditory, VA/SN and DMN predefined regions of interest (ROI; Figure 1) and then entered into a first level analysis wherein the correlation coefficient of each ROI to all other ROIs was calculated. Resulting correlation coefficients were Fisher *z*-transformed. Each participant’s first level ROI-to-ROI connectivity matrices were then entered into a second level GLM to obtain group level estimates for connection-based and network-based inferences.

Functional network connectivity (FNC) analysis (Jafri et al., 2008) was used to ascertain if the expected anticorrelation between the VA/SN and DMN was weaker in the ADHD group relative to controls (O1). FNC analysis outputs an *F*-statistic representing the difference in network-level connectivity between groups and the significance of the *F*-statistic was corrected for by way of a false-discovery rate (FDR) cluster-level threshold of *p* < 0.05. Eventual *post hoc*, ROI-level exploration of the hypothesized reduced anticorrelation (ADHD > Controls) between the two networks utilized an uncorrected, one-tailed *p* < 0.05 connection-level threshold. In addition, for each network, an FDR corrected (*p* < 0.05) ROI-to-ROI, 2-tailed univariate analysis (Benjamini and Hochberg, 1995) was used to assess if there were differences in within-network FC between groups.

Seed-based ROI-to-ROI analysis explored the hypothesis that auditory regions would be more positively coupled with ROIs of the SN and less anticorrelated with ROIs of the DMN if ADHD participants were more inherently sensitive to their auditory environment (O2). To this end, a separate one-way MANOVA for each target ROI (VA/SN = 7 targets, Figure 1A; DMN = 4 targets; Figure 1B) was conducted to determine if there were group differences (ADHD > Controls) in FC with the 10 auditory seeds (Figure 1C). Thus, for each target ROI, the connectivity values for all 10 seed-to-target pairs were entered as dependent variables in the MANOVA. To correct for multiple analyses, we utilized an FDR-adjusted significance threshold of *p* < 0.05. We additionally performed an FDR corrected (*p* < 0.05) ROI-to-ROI, 2-tailed univariate analysis (Benjamini and Hochberg, 1995) to assess whether group differences in auditory FC alone, were evident in our sample.

To explore the brain-behavior relationship between individual differences (collapsed across groups) in connectivity and 2b-T accuracy and ADHD-symptom severity (O3), a threshold free cluster enhancement (TFCE) procedure (Smith and Nichols, 2009) was used to correct for multiple comparisons. First, the connectivity maps, made up of the 210 possible ROI-to-ROI pairs pertaining to the 21 ROIs of the DMN, VA/SN and auditory regions collectively, were sorted using a hierarchical optimal leaf ordering procedure (Bar-Joseph et al., 2001) embedded in the CONN-toolbox. CONN-toolbox’s default statistical settings for TFCE analysis were then applied to identify significant clusters of ROI-to-ROI connections associated with 2b-T accuracy as well as symptom severity. This resulted in a TFCE score for each cluster and a family-wise error (FWE) corrected threshold of *p* < 0.05 (estimated using 1,000 permutation iterations of the data) was used to determine the significance of the TFCE scores. Eventual *post hoc* analysis utilized an uncorrected, two-tailed *p* < 0.05 connection-level threshold to identify the individual within-cluster ROI-to-ROI connections associated with the brain-behavior relationship. 2b-T data was missing for one of the ADHD participants, so this TFCE analysis included only 16 of the 17 ADHD participants. Similarly, impulsivity scores were missing for one of the control participants so TFCE analysis of the relationship between FC and impulsivity as well as total ASRS scores included only 16 of the 17 control participants.

## Results

### Group differences in default-mode network and ventral attention/salience network connectivity

Results of the FNC analysis confirmed that the anticorrelation between the DMN and VA/SN was significantly weaker *F*_(2, 31)_ = 7.2, *p*-FDR = 0.008, in ADHD than controls (O1). *Post hoc* analysis of ROI-to-ROI connections indicated that the weaker anticorrelation (*p*-uncorrected) was most strongly associated with the medial prefrontal cortex (PFC) of the DMN and was evident across all VA/SN regions (Table 1A). The posterior cingulate cortex (PCC) of the DMN also contributed to the weaker anticorrelation of which coupling included the left anterior insula, the anterior cingulate and bilateral rostral PFC of the VA/SN (Table 1A). As an additional, *post hoc* explorative step, we tested to see if any of these DMN-VA/SN connections correlated positively (one-tailed, Spearman’s rho) with individual differences (collapsed across groups) in ADHD-symptom severity. Results indicated that the weaker anticorrelated DMN–VA/SN FC was mostly associated with the severity of inattentive and combined symptoms across participants (see Table 1B for details). Tests for group differences in within-network FC were not significant.

**TABLE 1:** **(A)** Results of the *post hoc* analysis (one-tailed, independent *t*-tests) characterizing the individual default-mode network–ventral attention/salience network (DMN–VA/SN) connections that were significantly (*p* < 0.05, uncorrected) less anticorrelated in attention deficit hyperactivity disorder (ADHD) participants compared to controls. **(B)** Results of the explorative *post hoc* correlation analysis (Spearman’s rho) assessing the relationship of ADHD-symptom severity (inattentive, impulsive/hyperactive, combined) with the degree of reduced anticorrelated VA/SN–DMN functional connectivity (FC) across participants.

		A		B		
DMN –	VA/SN	*t* (32)	*p*	Inattentive	Impulsive	Combined
Medial PFC	Anterior insula R	3.5	0.001	0.51**	0.41**	0.50**
	Rostral PFC R	3.0	0.006	0.43**	0.39*	0.48**
	Supramarginal gyrus R	2.9	0.007	0.33*	0.22	0.31*
	Anterior cingulate	2.8	0.009	0.37*	0.22	0.37*
	Supramarginal gyrus L	2.6	0.015	0.38*	0.36*	0.39*
	Anterior insula L	2.3	0.029	0.35*	0.24	0.31*
	Rostral PFC L	2.0	0.030	0.28	0.19	0.29
Posterior cingulate	Anterior insula L	2.7	0.006	0.45**	0.11	0.29
	Anterior cingulate	2.4	0.012	0.37*	0.19	0.30*
	Rostral PFC L	2.2	0.017	0.39*	0.17	0.32*
	Rostral PFC R	1.8	0.043	0.32*	0.15	0.29

Asterik indicate significant rho-values (*p < 0.05, **p < 0.01, ***p < 0.001). L, Left; R, right; PFC, prefrontal cortex.

### Group differences in auditory connectivity

Between-group, seed-based analysis indicated that the phasic resting-state activity in auditory regions was significantly more positively correlated with the right supramarginal gyrus (SMG) of the VA/SN, *F*_(10, 23)_ = 4.7, *p*-FDR = 0.006; and significantly less anticorrelated with the medial PFC of the DMN *F*_(10, 23)_ = 4.8, *p*-FDR = 0.006, in the ADHD group; providing support to the hypothesis that adults with ADHD may be more inherently sensitive to their acoustic environment (O2). No differences between groups were observed for any of the other target ROIs. Nor did we observe groups differences in FC between auditory regions alone.

### Individual differences in brain-behavior associations

**FIGURE 2:**
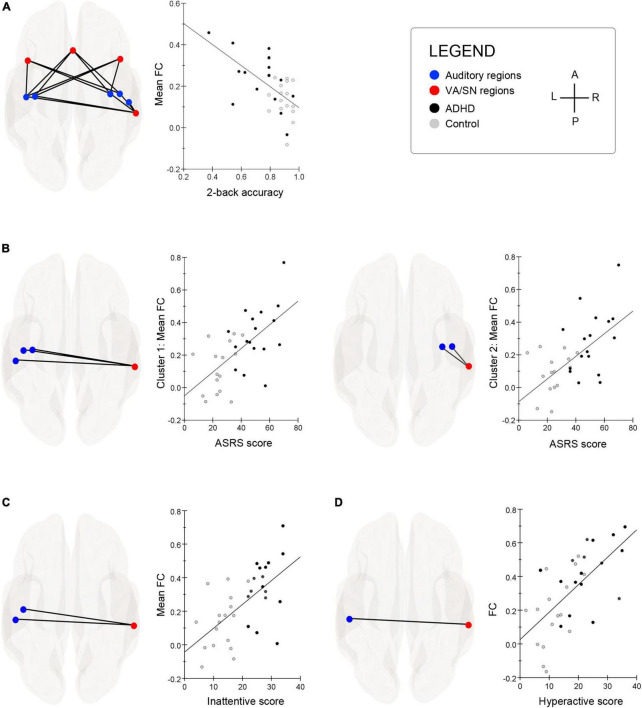
**(A)** Superior glass-brain images display the auditory–ventral attention/salience network (VA/SN) connections that were significantly associated with 2-back task (2b-T) accuracy. Scatter-chart shows that the more segregated the resting-state VA/SN–auditory functional connectivity (FC), the better the individual performed on a 2b-T whilst ignoring a distracting acoustic signal (for visualization purposes, *y*-axis represents the average of all significant FC values associated with 2b-T accuracy). **(B)** The threshold free cluster enhancement (TFCE) procedure identified two significant clusters of connections between the right supramarginal gyrus (SMG) and auditory ROIs that were positively associated with combined ADHD-symptom severity scores (ASRS). Cluster 1: left glass-brain; Cluster 2: right-glass brain. Associated scatter-charts show that increased resting-state FC between the right SMG and auditory ROIs was correlated with attention deficit hyperactivity disorder (ADHD)-symptom severity (*y-*axis represents the average of all significant FC values within each cluster). **(C)** Hyperconnectivity between the right SMG and left lateralized Heschl’s gyrus and planum temporale was positively associated with participants’ inattentive scores. Scatter chart depicts the strength of relationship collapsed across groups (*y-*axis represents the average of all significant FC values within each cluster). **(D)** Hyperconnectivity between the right SMG and left lateralized planum temporale was positively associated with participants’ hyperactivity/impulsivity scores. Scatter chart depicts the strength of relationship collapsed across groups. A, anterior; P, posterior; L, left; R, right.

The TFCE clustering procedure (O3), identified a significant cluster of connections between auditory and VA/SN ROIs that were negatively associated with 2b-T accuracy. TFCE = 34.9, *p*-FWE = 0.040. *Post hoc* connection-level analysis (see Table 2 for detailed statistics) indicated that the negative brain-behavior relationship mostly involved FC between early auditory processing regions and core hubs of the VA/SN: the anterior cingulate, the anterior insulae and the right SMG (Figure 2A). Taken together, these results suggest that individuals who could perform well on the cognitively demanding working memory task whilst ignoring distracting acoustic stimulation also had more intrinsically segregated auditory–VA/SN connectivity at rest. This pattern of FC was also shown to have an inverse relationship with ADHD-symptom severity. TFCE analysis identified two significant clusters of auditory-VA/SN connections that were positively associated with symptom severity (Figure 2B). Cluster one, TFCE = 58.3, *p*-FWE = 0.006, consisted of increased FC between the right SMG and left lateralized auditory ROIs; and cluster two, TFCE = 40.07, *p*-FWE = 0.035, consisted of increased FC between the right SMG and right lateralized auditory ROIs (see Table 2 for detailed connection-level statistics). TFCE analysis additionally identified one FC cluster, TFCE = 44.8, *p*-FWE = 0.032 that correlated with inattentive scores (Figure 2C) and one FC cluster, TFCE = 50.5, *p*-FWE = 0.014 that correlated with impulsivity scores (Figure 2D) wherein increases in inattentiveness and impulsivity were both associated with increased FC between the right SMG and left lateralized auditory ROIs.

**TABLE 2:** Table lists connection-level results for the clusters of VN/SN–auditory connections that were negatively associated with 2b-T accuracy and positively associated with attention deficit hyperactivity disorder (ADHD)-symptom severity (combined, inattentiveness and impulsivity). One-sample *t*-values, indicate that the correlation was significantly (*p* < 0.05, uncorrected, two-tailed) different from zero. Pearson’s correlation coefficient (*r*) indicates the strength of the relationship with the behavioral variable for each ROI-to-ROI connection.

2-back task accuracy	VA/SN -	Auditory	*t* (31)^†^	*p*	*r*
	Anterior cingulate	Heschl’s gyrus L	−2.6	0.014	−0.42
		Granular posterior insula L	−2.1	0.045	−0.37
		Heschl’s gyrus R	−2.1	0.045	−0.35
		Granular posterior insula R	−3.1	0.004	−0.48
	Anterior insula L	Heschl’s gyrus L	−2.6	0.014	−0.42
		Heschl’s gyrus R	−3.3	0.003	−0.51
		Granular posterior insula R	−3.5	0.001	−0.53
	Anterior insula R	Heschl’s gyrus L	−2.6	0.014	−0.42
		Granular posterior insula L	−2.2	0.034	−0.37
		Granular posterior insula R	−2.8	0.009	−0.44
	Supramarginal gyrus R	Heschl’s gyrus L	−2.5	0.018	−0.41
		Granular posterior insula L	−2.7	0.013	−0.43
		Heschl’s gyrus R	−2.2	0.035	−0.37
		Planum temporale R	−2.1	0.045	−0.35
		Granular posterior insula R	−2.8	0.010	−0.44

**ADHD combined**	**VA/SN -**	**Auditory**	***t* (31)^**‡**^**	***p***	***r***

Cluster 1:	Supramarginal gyrus R	Heschl’s gyrus L	3.7	0.001	0.55
		Granular posterior insula L	3.4	0.002	0.53
		Planum temporale L	4.7	0.000	0.65
Cluster 2:	Supramarginal gyrus R	Granular posterior insula R	4.1	0.000	0.59
		Heschl’s gyrus R	3.0	0.006	0.47

**ADHD combined**	**VA/SN -**	**Auditory**	***t* (31)^**‡**^**	***p***	***r***

	Supramarginal gyrus R	Heschl’s gyrus L	3.8	0.000	0.55
		Planum temporale L	4.2	0.001	0.59

**ADHD combined**	**VA/SN -**	**Auditory**	***t* (31)^**‡**^**	***p***	***r***

	Supramarginal gyrus R	Planum temporale L	4.7	0.000	0.64

^†^Analysis conducted on 33 of the 34 participants because 2b-T data was missing for one of the ADHD participants.

^‡^Analysis conducted on 33 of the 34 participants because impulsivity scores were missing for one the control participants.

L, left; R, right.

## Discussion

The purpose of the current study was to explore whether adults with ADHD would show aberrant FC compared to healthy controls between DMN, VA/SN, and auditory regions. Our combined results were rather striking. First, as per expectations, a weaker anticorrelation between the VA/SN and DMN was observed in ADHD participants. Second, this aberrant connectivity was underscored by an enhanced coupling between auditory ROIs and the right SMG of the VA/SN and a reduced anticorrelation between the medial PFC of the DMN and auditory ROIs. Third, it was shown that participants who displayed strong intrinsic segregation of the VA/SN and auditory ROIs at rest, were also better at performing well on a cognitively demanding visual working memory task that concurrently required participants to ignore a streaming acoustic signal. We discuss these results in more detail over the proceeding subsections.

### Group differences in functional network connectivity

We observed a reduced anticorrelation between regions of the VA/SN and DMN in our sample of ADHD-adults relative to controls. This finding is in line with several previous studies (e.g., Castellanos et al., 2008; Sun et al., 2012; Sripada et al., 2014; Lin et al., 2018; Mills et al., 2018; c.f. also Sato et al., 2012) and contributes further evidence for an impaired functional organization between these two networks in the disorder. Particularly noteworthy, is that our results replicate the findings of Lin et al. (2018, Supplementary Figure 4), who used the same FNC analysis (Jafri et al., 2008) we employed here in a large medication-naive sample (*n* = 80) of adults with ADHD. However, unlike some reports from previous studies in ADHD (see Castellanos and Aoki, 2016 for a review), we did not observe reduced FC-strength between ROIs of the DMN in ADHD participants. Although it is difficult to speculate why we did not replicate this finding our sample, the fact that we did not find group differences in within-network FC for either the DMN or the VA/SN suggests that the weaker between network anticorrelation was unlikely being driven by a single, abnormally regulated network.

Individual differences in the frequency of inattentive and combined symptoms also correlated with the magnitude of reduced anticorrelation between the VA/SN and DMN across participants. Interestingly, the *default-mode interference* hypothesis (Sonuga-Barke and Castellanos, 2007) has tended to dominate interpretations of this atypical connectivity associated with symptomatic inattentiveness in ADHD, wherein the weaker anticorrelation is conceived as an index for spontaneous mind wandering (cf, Kelly et al., 2008). However, an alternative hypothesis suggests that a reduced anticorrelation may additionally index an inherent susceptibility to environmental distraction (Menon, 2011; Aboitiz et al., 2014). Consistent with this latter hypothesis, we also observed in the ADHD group, an enhanced coupling between auditory regions and the right SMG. The SMG is an integrative hub of the VA/SN, and the right lateralized region has been particularly implicated in the mediation of exogenous attention toward visual, tactile and auditory modalities (Corbetta and Shulman, 2002; Corbetta et al., 2008; Cabeza et al., 2012; Vossel et al., 2014). The increased FC between the VA/SN and auditory regions may mean that the right SMG is intrinsically biased to the auditory modality in ADHD and indicative of a symptomatic, heightened sensitivity to the acoustic environment. Alternatively, the increased connectivity may be indicative of increased functional communications between the right SMG and auditory ROIs which would suggest that participants with ADHD were more aware of their acoustic environment throughout the resting-state. Further experimental research is needed to disentangle these two alternatives, but either way, both alternatives lend support to the hypothesis that adults with ADHD are more susceptible to auditory distraction.

Interestingly, a similar notion was underscored in an original study by Schulze et al. (2021) that explored whether aberrant intrinsic network FC was associated with performance deficits in multisensory integration in adult ADHD. By way of the McGurk illusion, the authors found that ADHD participants experienced significantly fewer illusions to that of healthy controls due to a sensory bias for auditory stimuli. And how well participants integrated the McGurk illusion was negatively associated with childhood symptom severity and self-rated inattentiveness in adulthood. Resting-state FC in ADHD participants was increased between the planum temporale and anterior insula for which the authors speculated that this auditory–VA/SN hyperconnectivity may be indicative of an increased susceptibility to auditory distraction. Interestingly, performance on the McGurk illusion was inversely associated with hyperconnectivity between the Heschl’s gyrus and the middle temporal gyrus (a convergence zone for e.g., audiovisual integration). Taken together, Schulze et al. (2021) concluded that communication between sensory areas to integrative hub regions might be disrupted in ADHD, impacting the appropriate assignment of top-down attentional allocation.

The current study also observed a reduced anticorrelation between auditory regions and the medial PFC in our ADHD sample relative to controls. The medial PFC is a highly metabolically demanding hub of the DMN and similarly to the VA/SN, has been shown to have an anticorrelated relationship with sensory systems (e.g., Greicius and Menon, 2004). Interestingly, our finding is consistent with a study by Cocchi et al. (2012) who observed a reduced anticorrelation between the medial PFC of the DMN and an associative auditory region in the left superior temporal lobe in their non-clinical sample of drug-naive young adult students with ADHD. Our sample was similar to that of Cocchi et al.’s (2012) in that the majority of participants from both control and clinical groups were young adult students. The fact that Cocchi et al. (2012) observed similarly altered auditory–DMN FC in their drug-naive sample, that additionally correlated with ADHD-symptom severity scores, reinforces the hypothesis that the DMN is a locus of dysfunction in ADHD (see for example: Castellanos et al., 2009). However, Cocchi et al.’s (2012) findings, in combination with our own, are also an indication that the behavioral implication of DMN dysfunction in ADHD is not limited to attentional lapses associated with spontaneous mind wandering. Indeed, although heavily implicated in internal mentation (Gusnard et al., 2001), additional documented functions for the medial PFC include passive monitoring of the environment (Buckner et al., 2008; Dohmatob et al., 2017), perceptual binding (Martínez-Sanchis, 2014) and top-down modulation of sensory interference (Kucyi et al., 2013; Chadick et al., 2014; Martin-Cortecero and Nuñez, 2016). Collectively, these additional functions suggest that the DMN also plays an important role in both sensory and attentional processing and provide important clues about the implications that aberrant auditory–DMN functional organization may have with respect to auditory distraction in ADHD.

### Brain-behavior relationships

Our most striking finding was a resting-state relationship with performance from a demanding visual 2-back task (2b-T) that concurrently required participants to ignore a streaming acoustic signal (∼75 dB SPL). Throughout this task, controls were more proficient than ADHD participants at attenuating the evoked auditory activity from the distracting acoustic signal and performed overall better than ADHD participants. In addition, the more attenuated the auditory evoked responses were across participants, the lower their symptom severity scores of inattentiveness. In the current study, we showed that participants who had the capacity to perform well on the 2b-T whilst ignoring the distracting acoustic signal, also tended to have more intrinsically segregated VA/SN–auditory connectivity at rest. This pattern of VA/SN–auditory FC was also shown to have an inverse relationship with ADHD-symptom severity. Specifically, increased connectivity between the right SMG of the VA/SN and auditory cortices (Heschl’s gyrus, posterior insula, and planum temporale) was positively associated with the severity of ADHD-symptoms across participants. In sum, the resting-state brain-behavior relationships observed in the current study are supportive of theories that suggest that the VA/SN plays a pivotal role in the manifestation of attentional deficits in neuropsychiatric disorders (Menon, 2011), wherein an impaired intrinsic organization of the VA/SN can lead to an inappropriate bias toward irrelevant/salient stimuli, and behaviorally, result in an increased susceptibility for environmental distraction.

### Limitations

Because we have already addressed in detail the limitations regarding our sample in Blomberg et al. (2021), these will only be briefly listed here. First, our ADHD-sample included more females than males. Although childhood-ADHD is more commonly diagnosed in boys, the differences in prevalence between sexes diminishes almost completely in adulthood (Faraone et al., 2015; Matte et al., 2015), so we should not expect the gender imbalance in our sample to dramatically affect more general conclusions of our results. Second, our ADHD-sample included medicated individuals, half of which were also on stable SSRI medication, which is indicative of earlier problems with anxiety and depression. However, both anxiety and depression are at the lower end of the spectrum of expected psychiatric comorbidities in adults with ADHD (Katzman et al., 2017). If individuals with ADHD are expected to have more severe symptoms and functional impairment than our sample, then the group differences reported here are likely to underestimate the overall differences between groups in the general population, rendering our results conservative. In addition, it is worth noting that similar findings of aberrant resting-state connectivity associated with ADHD-symptoms reported in the current study, have also been observed in other studies with medication-naive adult samples (Cocchi et al., 2012; Lin et al., 2018).

In addition to the aforementioned limitations regarding our sample, two methodological limitations specific to this study are worthy of addressing. First, we did not implement any physiological methods that would allow us to monitor the arousal levels of the participants throughout the resting-state period. The functional resting-state duration was ∼12 min—prior to which participants also underwent ∼6 min of anatomical scans—and it is known that some participants can fall asleep in the scanner under conditions where they are left to rely on mentation as their sole source of stimulation. We can however, state that only two accepted applicants for this study reported falling asleep in the scanner (one ADHD and one control), and these participants were thereby excluded from the sample used here and in Blomberg et al. (2021). Because our main purpose was to explore whether adults with ADHD are inherently more sensitive to their acoustic environment—which to some degree involves a heightened level of awareness to auditory stimulation—our findings are still interesting, even if participants waned in their levels of arousal throughout the resting-state period.

A second limitation is that we did not have a means of monitoring eyes-open versus eyes-closed in participants. Participants wore MRI goggles in the scanner throughout the anatomical and functional scan. Because the anatomical scan did not require participants to have their eyes closed, the goggles presented a dimly lit word (dark gray on black background) reading: *REST* (*VILA* in Swedish), which slowly moved (figure-eight animation) within participants’ field of view. This animation continued throughout the duration of the resting-state period. Had participants opted to open their eyes during the resting-state scan, then this was the only visual stimulation they received. Compared with a visually salient fixation cross—which many studies use under these circumstances—we tentatively suggest that our choice of visual stimulation, albeit novel, may have at least circumvented unwanted externally-directed frontal eye field activity associated with ocular fixation (Vernet et al., 2014), even though visual cortical activity was imminent. Eyes-open resting-state is known to result in increased visual network connectivity (Yang et al., 2007). However, the modality of interest in the current study was the auditory modality, so we should not expect that eventual periodic states of eyes-open to impact the study’s overall conclusions.

## Conclusion

In accord with previous studies, a reduced resting-state anticorrelation between the VA/SN and DMN was observed in our sample of adults with ADHD. Moreover, core hubs of the DMN and VA/SN, which respectively have been implicated in top-down and bottom-up regulation of attention to sensory events, exhibited aberrant FC with the auditory network in ADHD participants. Additionally, it was shown that participants who displayed stronger intrinsic segregation of the VA/SN and auditory network at rest, were also better at performing well on a cognitively demanding visual working memory task whilst attenuating distracting auditory stimulation (task conditions where ADHD participants proved inferior to controls). Overall, our collective results are consistent with the hypothesis that auditory distraction and more generally inattentiveness in ADHD is linked to aberrant interactions between DMN, VA/SN, and auditory systems. Importantly, our findings contribute further evidence to current etiological models of ADHD that implicate dysfunctional organization of DMN, VA/SN and other major intrinsic networks in behavioral and clinical symptoms in the disorder. Our findings also encourage more research into sensory interactions with these major intrinsic networks so that we can refine our theories of inattention and better understand factors that impact symptoms of sensory distraction in the disorder.

## Data availability statement

The raw data supporting the conclusions of this article will be made available by the authors, without undue reservation.

## Ethics statement

The studies involving human participants were reviewed and approved by the Regional Ethical Review Board in Linköping, Sweden (DNR 2019-06158). The patients/participants provided their written informed consent to participate in this study.

## Author contributions

JR, RB, AC, and HD contributed to the conception and design of the study. AC and RB were responsible for participant recruitment and data collection. RB was responsible for data analysis as well as manuscript drafting and was assisted by IP, AC, and CS. All authors scrutinized the statistical analysis, contributed to the manuscript’s revision, and approved submitted version.

## Footnotes

1 www.nitrc.org/projects/conn, RRID:SCR_009550
