# Supplementary material for: Aberrant resting-state connectivity of auditory, ventral attention/salience and default-mode networks in adults with attention deficit hyperactivity disorder
Source: Front Neurosci. 2022 Sep 6;16:972730.. doi: 10.3389/fnins.2022.972730 (PMC9485623; doi:10.3389/fnins.2022.972730)
Supplement: Supplementary file 1 [file Data_Sheet_1.PDF]

Supplementary methods

DEMOGRAPHIC AND CLINICAL DETAILS OF THE SAMPLE

The resting state data for this study was obtained from the sample of participants from Blomberg et al. (2021). Adults volunteered for the study through advertisements posted in campus, clinics, and online social media platforms. To assess inclusion criteria, all applicants were required to fill in a digital questionnaire regarding age, health, handedness, alcohol/substance use, diagnosis/es and medications. Applicants were excluded if they did not meet age requirements (18-50 *yrs*); were dominantly left-handed; had medical/psychiatric conditions/disabilities that could affect the quality of the data (e.g., severe acute psychiatric disorders; ASD or hearing loss); reported frequent use of alcohol/substances; or were medicated with medication that could affect arousal levels (e.g., neuroleptics, sedatives, and/or opioids). In addition, applicants with an ADHD diagnosis were only included if either medicating with central stimulants and prepared to undergo a 48 *hrs* washout period prior to testing or were currently unmedicated for their ADHD.

Outside of the scanner and in a quiet room, clinical assessments of attention, ADHD-symptom severity, and the presence of comorbid disorders and problems with substance abuse were investigated in more detail by way of the d2-R Test of Attention (Brickenkamp et al., 2010), the 18-item Adult ADHD Self-Report Scale (ASRS) v.1.1 (Kessler et al., 2005; Rodriguez et al., 2007) and the Mini-International Neuropsychiatric Interview (MINI) 7.0.2 DSM-5 for ADHD studies (Sheehan et al., 1998) respectively. Individual scores for the ASRS were calculated by summing scores from items associated with self-reported attentional difficulties (Part A: 1 – 4; Part B: 1 – 5), hyperactivity/impulsivity problems (Part A: 5 – 6; Part B: 6 – 12), as well as the sum of all 18 items as a general index of symptom severity. Standard scoring procedures were used for each index in the d2-R and MINI. Pure-tone audiometry at six frequencies ranging from 0.25 *kHz* to 8 *kHz* was also used to screen participants for normal (< 20 *dB* HL) hearing thresholds (in accordance with: American Speech-Language-Hearing Association, 2005). Participants also completed two complex working span tests: the Size-comparison span test (Sörqvist et al., 2010) as a measure of both working memory gating and maintenance; and the Reading span task (Rönnberg et al., 1989) to measure working memory maintenance, procedures for which are published in Blomberg et al. (2019). See Table S1 for more descriptive details of the sample.

Table S1 Demographic and clinical details of the sample.

	Controls (n = 17)	ADHD (n = 17)	Group comparisons
**Current/highest education level**	***N***	***N***	
Upper secondary	1	4	
Undergraduate	14	13	
Postgraduate	2	-	
**Hearing acuity (dB)**	***M* (*SD*)**	***M* (*SD*)**	***F*-test**
Pure-tone average	-2.5 (4.1)	-0.3 (7.4)	*Welch's* $F(1, 25.3) = 1.4$, *ns*
**d2-Test Attention (standard score)**	***M* (*SD*)**	***M* (*SD*)**	***F*-test**
Concentration	103 (7.1)	100 (9.0)	*Welch's* $F(1, 30.4) = 1.3$, *ns*
Processing speed	103 (15.6)	106 (15.2)	$F(1, 32) = .31$, *ns*
Precision	100 (9.9)	97 (11.6)	$F(1, 32) = .63$, *ns*
**ASRS v.1.1 (aggregate score)**	***M* (*SD*)**	***M* (*SD*)**	***F*-test**
Inattention	12.8 (4.7)	27.5 (3.9)	$F(1, 32) = 97.1, p < .000$
Impulsivity	11.9ᵻ (5.7)	23.0 (8.1)	$F(1, 31) = 20.6, p < .000$
Combined	24.6ᵻ (9.2)	50.5 (11.5)	$F(1, 31) = 49.5, p < .000$
**MINI 7.0.1 for ADHD studies**	***N***	***N***	
Inattentive	1	6	
Impulsive	-	-	
Combined	-	11	
**Working memory span**	***M* (*SD*)**	***M* (*SD*)**	***F*-test**
Size-comparison span	.51 (.12)	.46 (.16)	*Welch's* $F(1,24.8) = 1.4$, *ns*
Reading span	.71 (.12)	.63 (.23)	$F(1,32) = 1.3$, *ns*

ᵻ *Mean derived from 16 out of the 17 Controls as item 8 (Part B) was missing for one of the participants.*

SUPPLEMENTARY REFERENCES

American Speech-Language-Hearing Association. (2005). *Guidelines for manual pure-tone threshold audiometry [Guidelines]*. https://doi.org/doi:10.1044/policy.GL2005-00014

Blomberg, R., Johansson Capusan, A., Signoret, C., Danielsson, H., & Rönnberg, J. (2021). The Effects of Working Memory Load on Auditory Distraction in Adults With Attention Deficit Hyperactivity Disorder. *Frontiers in Human Neuroscience*, *15*, 724. https://doi.org/10.3389/fnhum.2021.771711

Brickenkamp, R., Schmidt-Atzert, L., & Liepmann, D. (2010). *D2-R*. Hogrefe.

Kessler, R. C., Adler, L., Ames, M., Demler, O., Faraone, S., Hiripi, E., Howes, M. J., Jin, R., Secnik, K., Spencer, T., Ustun, T. B., & Walters, E. E. (2005). The World Health Organization adult ADHD self-report scale (ASRS): A short screening scale for use in the general population. *Psychological Medicine*, *35*(2), 245–256. https://doi.org/10.1017/S0033291704002892

Rodriguez, A., Ginsberg, Y., Fernholm, A., & Nyberg, L. (2007). ADHD difficult to diagnose in adults. ASRS v1.1 Self-Report Scales valuable help—Now translated to Swedish. *Lakartidningen*, *104*(18), 1398–1400.

Rönnberg, J., Arlinger, S., Lyxell, B., & Kinnefors, C. (1989). Visual evoked potentials: Relation to adult speechreading and cognitive function. *Journal of Speech and Hearing Research*, *32*(4), 725–735.

Sheehan, D. V., Lecrubier, Y., Sheehan, K. H., Amorim, P., Janavs, J., Weiller, E., Hergueta, T., Baker, R., & Dunbar, G. C. (1998). The Mini-International Neuropsychiatric Interview (M.I.N.I.): The development and validation of a structured diagnostic psychiatric interview for DSM-IV and ICD-10. *The Journal of Clinical Psychiatry*, *59 Suppl 20*, 22-33;quiz 34-57.

Sörqvist, P., Ljungberg, J. K., & Ljung, R. (2010). A sub-process view of working memory capacity: Evidence from effects of speech on prose memory. *Memory*, *18*(3), 310–326. https://doi.org/10.1080/09658211003601530
